# Role of Somatostatin in Preventing Post-endoscopic Retrograde Cholangiopancreatography (ERCP) Pancreatitis: An Update Meta-analysis

**DOI:** 10.3389/fphar.2016.00489

**Published:** 2016-12-15

**Authors:** Jing Hu, Pei-Lin Li, Tao Zhang, Jin-Ping Chen, Yao-Jun Hu, Zheng Yu, Jin-Peng Wang, Dan Zhu, Xiao-Fei Tong

**Affiliations:** Department of Gastroenterology, Fu Xing Hospital, Capital Medical UniversityBeijing, China

**Keywords:** somatostatin, endoscopic retrograde cholangiopancreatography, pancreatitis, hyperamylasemia, meta-analysis

## Abstract

**Background:** Acute pancreatitis is the most common serious complication of endoscopic retrograde cholangiopancreatography (ERCP). Although, somatostatin (SOM) has been used in the prevention of post-ERCP pancreatitis (PEP), the efficacy of SOM remains inconsistent.

**Methods:** Electronic databases, including PubMed/MEDLINE, EMBASE, the Cochrane Central Register of Controlled Trials (CENTRAL, The Cochrane Library), and the Science Citation Index were searched to retrieve relevant studies. Details of the study population, including patients’ characteristics, sample size, regimen of drug administration and incidence of PEP, hyperamylasemia and abdominal pain were extracted by two investigators. Data were analyzed with Review Manager 5.3 software.

**Results:** Eleven randomized controlled trials, enrolling a total of 4192 patients, were included in the meta-analysis. After data were pooled, we observed decreased incidence of ERCP-induced outcomes, such as PEP (RR = 0.63, 95% CI: 0.40, 0.98; *P* = 0.04) and hyperamylasemia (RR = 0.75, 95% CI: 0.66, 0.84; *P* < 0.001) in patients treated with SOM than those with placebo. Subgroup analysis by ethnicity found decreased incidence of PEP and hyperamylasemia in Asia only. Subgroup analysis by treatment schedule and dosage revealed decreased incidence of PEP and hyperamylasemia when SOM were treated with a single bolus or long-term infusion, or at dose above 3000 μg. We did not observed efficacy of SOM on abdominal pain in pooled or subgroup analysis.

**Conclusion:** This meta-analysis of patients undergoing ERCP showed reduced incidence of PEP and hyperamylasemia when SOM was administrated with single bolus, long-term infusion, or high dosage. More data are needed to confirm our findings.

## Introduction

Acute pancreatitis is the most common serious complication of endoscopic retrograde cholangiopancreatography (ERCP) that has been associated with high morbidity and mortality (Freeman and Guda, 2004). It occurs in 2–9% general population and 15% in high-risk patients (Cheng et al., 2006). Although, mild post-ERCP pancreatitis (PEP) generally has few complications with optimal outcome, severe PEP can lead to serious complications, such as systemic inflammatory response, pseudocysts or pancreatic necrosis and even death in a significant portion of patients. In the last three decades, extensive studies were made to investigate the solutions of reducing associated risk and increasing the safety (Choudhary et al., 2011; Dumonceau et al., 2014; Luo et al., 2016). It has demonstrated that the major therapy for PEP is pharmaco-prevention with different agents, such as somatostatin (SOM), non-steroidal anti-inflammatory drugs (NSAIDs), and indomethacin (Kubiliun et al., 2015).

Somatostatin, a potent inhibitor of pancreatic exocrine function, has been found to prevent or mitigate the processes of pancreatic inflammation. However, the efficacy is not consistent when SOM was used at different doses and duration schedules. Rudin et al. (2007) found that SOM administered as a bolus could reduce the incidence of ERCP-induced complications, such as PEP and hyperamylasemia. Consistently, a meta-analysis reported decreased risk of PEP in patients receiving high-dose SOM over 12 h (Omata et al., 2010). A recent meta-analysis further demonstrated decreased incidence of PEP at a single bolus or long-term injection, but no decreased incidence when given as short-term infusion, but (Qin et al., 2015). Therefore, further investigation is required to uncover appropriate methods of administration of SOM to prevent PEP.

In this study, we aim to reassess the effects of SOM on ERCP-induced complications, including PEP, hyperamylasemia and abdominal pain using a meta-analytic approach. We also performed subgroup analyses according to ethnicity, treatment schedule, and dosage to investigate the rational application of SOM for improved benefits.

## Materials and Methods

### Literature Search

Literature search was conducted according to the PRISMA statement developed specifically for meta-analyses to improve the reporting of reviews (Moher et al., 2009). The following databases were searched from their inception through July 2016: PubMed/MEDLINE, EMBASE, the Cochrane Central Register of Controlled Trials (CENTRAL, The Cochrane Library), and the Science Citation Index. Search strategy was performed with both free-text terms and MeSH terms, including “pancreatitis,” “ERCP,” and “somatostatin.” There is no requirement on publication date or type of studies.

### Study Selection

Two investigators (Pei-Lin Li, Tao Zhang) independently reviewed titles and abstracts for relevance. All articles assessed as relevant were included for full-text review. The criteria for inclusion were: (1) randomized controlled trials (RCTs), (2) reporting at least two outcomes, (3) only the most recent study was included if more than one study was published using the same study population. Open or uncontrolled clinical trials, observational studies and case reports were excluded from the meta-analysis.

### Data Extraction and Quality Assessment

Two investigators (Tao Zhang, Jin-Ping Chen) independently extracted details of the study population, including patients’ characteristics, sample size, regimen of drug administration and incidence of PEP, hyperamylasemia and abdominal pain. The Jadad score is used to assess the methodological quality of selected studies (Jadad et al., 1996). Assessment discrepancies were resolved by discussion with by Dr. Jing Hu until consensus was reached.

### Data Analysis

Meta-analyses were conducted using Review Manager 5.3 (Cochrane Collaboration, Oxford, UK). Dichotomous data, such as incidence of PEP, hyperamylasemia and abdominal pain, were expressed as relative risk (RR) with 95% confidence interval (CI). Heterogeneity was quantified with Cochran’s *Q* test. When there was considerable heterogeneity (*P* ≥ 0.10 and *I^2^* ≤ 50%), the data were analyzed in a fixed-effects model. When there was low heterogeneity (*I^2^* > 50% or *P* < 0.10), the data were analyzed in a fixed-effects mode. A funnel plot was used to assess potential publication bias.

## Results

### Identification of Eligible Studies

**FIGURE 1:**
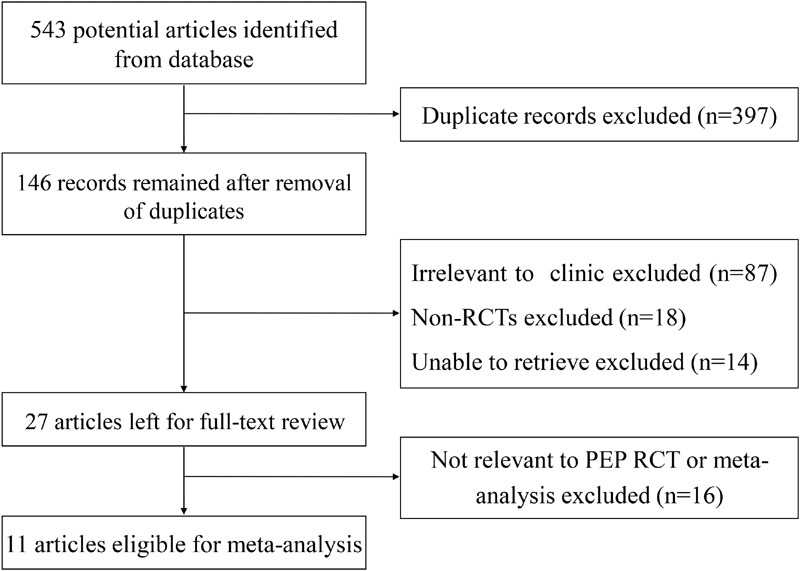
**Flow diagram of eligible articles selection process**.

As shown in **Figure 1**, a total of 543 articles were identified and 146 were remained after removal of duplications. 119 articles were excluded: 87 articles due to non-relevance, 18 non-RCT articles, and 14 articles unable to retrieve. In total, 27 articles were reviewed in detail, of which 16 were excluded due to non-relevance. Finally, 11 eligible studies satisfied the criteria for our meta-analysis.

### Characteristics of Included Studies

The baseline characteristics of the 11 studies published between 1998 to 2015 are presented in Supplementary Table S1 (Bordas et al., 1998; Poon et al., 1999; Andriulli et al., 2002, 2004; Poon et al., 2003; Arvanitidis et al., 2004; Chan et al., 2008; Lee et al., 2008; Wang et al., 2013; Concepción-Martín et al., 2014; Bai et al., 2015). Totally, 4192 patients undergo ERCP procedures above average age of 58 years were analyzed. The Jaded score for 11 RCTs were ≥4, suggesting their high quality. **Table 1** lists the intervention of SOM and post-ERCP complications.

**Table 1:** Intervention in 11 RCTs.

Study (years)	Treatment style of SOM	Dosage	Starting time of therapy	Duration	Outcome measures
Bordas et al., 1998	Intravenous injection	4 μg/kg	On identification of papilla	Bolus	IPEP
Poon et al., 1999	Intravenous injection	3000 μg	30 min before ERCP	12 h	IPEP, H, AP
Andriulli et al., 2002	Intravenous injection	750 μg	30 min before ERCP	2.5 h	IPEP, H, AP
Poon et al., 2003	Intravenous injection	250 μg	Immediately after diagnostic ERCP	Bolus	IPEP, H, AP
Andriulli et al., 2004	Intravenous injection	750 μg	30 min before ERCP	6.5 h	IPEP, H, AP
Arvanitidis et al., 2004	Intravenous injection	4 μg/kg	1 h before ERCP	Bolus	IPEP, H
		3000 μg		12 h	
Chan et al., 2008	Intravenous injection	250 μg/h	Before ERCP	Bolus plus 12 h	IPEP, H
		250 μg		Bolus	
Lee et al., 2008	Intravenous injection	3000 μg	30 min before ERCP	12 h	IPEP, H
Wang et al., 2013	Intravenous injection	250 μg/h	1 h before ERCP	24 h	IPEP, H
		250 μg/h	1 h after ERCP		
Concepción-Martín et al., 2014	Intravenous injection	1250 μg	Before cannulation of papilla	Bolus plus 4 h	IPEP, H, AP
Bai et al., 2015	Intravenous injection	3000 μg	Before ERCP	Bolus plus 11 h	IPEP, H

*SOM, somatostatin; ERCP, endoscopic retrograde cholangiopancreatography; IPEP, incidence of post-ERCP pancreatitis; H, hyperamylasemia; AP, abdominal pain.*

### Incidence of PEP

PEP occurred in 117 (5.44%) out of 2,152 patients treated with SOM, and 159 (7.79%) out of 2,040 patients treated with placebo (Supplementary Figure S1). The random-effect model demonstrated significantly decreased PEP risk in patients treated with SOM (RR = 0.63, 95% CI: 0.40, 0.98; *P* = 0.04).

**FIGURE 2:**
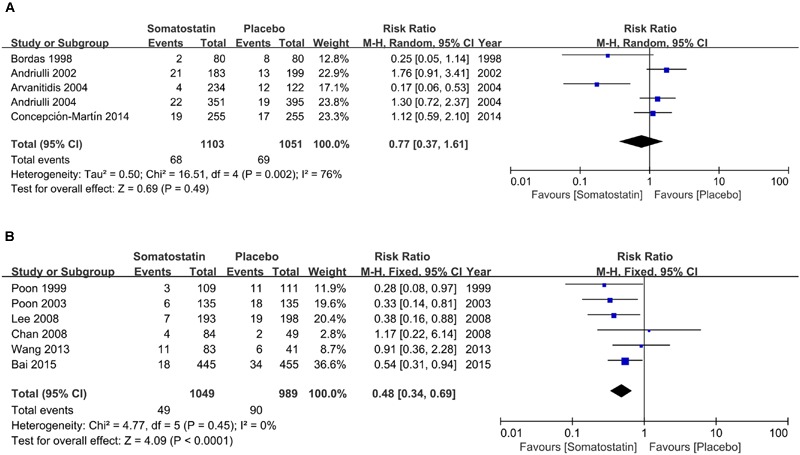
**Forest plot of RR of PEP in Europe (A)** and Asia **(B)**. *I*^2^ and *P* is the criterion of heterogeneity test, ◆ pooled RR, —■— RR and 95% CI.

We then performed subgroup analysis according to area, treatment schedule and dosage. In five RCTS in Europe, PEP incidence is comparable in patients treated with SOM (6.16%) and placebo (6.56%) (RR = 0.77, 95% CI: 0.37, 1.61; *P* = 0.49) (**Figure 2A**). In six RCTs in Asia, we found significantly decreased risk of PEP incidence when patients were treated with SOM (RR = 0.48, 95% CI: 0.34, 0.69; *P* < 0.001) (**Figure 2B**).

**FIGURE 3:**
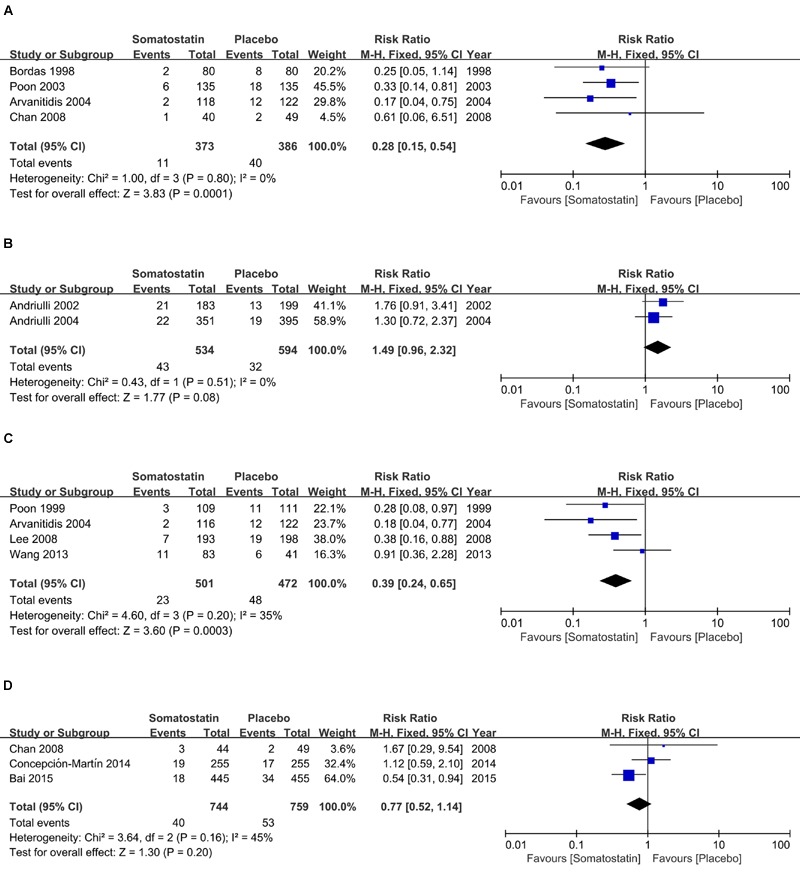
**Forest plot of RR of PEP in patients treated with SOM as single bolus (**A**)**, short-term infusion **(B)**, long-term infusion **(C)**, and bolus plus continuous infusion **(D)**. *I*^2^ and *P* is the criterion of heterogeneity test, ◆ pooled risk ratio, —■— risk ratio and 95% CI.

**Figure 3** shows RR in subgroup analysis according to doses and duration schedules of SOM. PEP risk is decreased in patients receiving SOM with a single bolus than placebo (2.95% vs. 10.36%) (RR = 0.28, 95% CI: 0.15, 0.54; *P* < 0.001) (**Figure 3A**). When SOM was administrated as short-term infusion, the incidence of PEP showed marginal decrease than placebo (8.05% vs. 5.39%) (R = 1.49, 95% CI: 0.96, 2.32; *P* = 0.08) (**Figure 3B**). We observed significantly decreased PEP risk when SOM was administrated as long-term infusion (RR = 0.39, 95% CI: 0.24, 0.65; *P* < 0.001) (**Figure 3C**). However, when SOM was treated as bolus plus continuous infusion, the incidence was comparable between SOM and placebo (5.38% vs. 6.98%) (RR = 0.77, 95% CI: 0.52, 1.14; *P* = 0.20) (**Figure 3D**).

**FIGURE 4:**
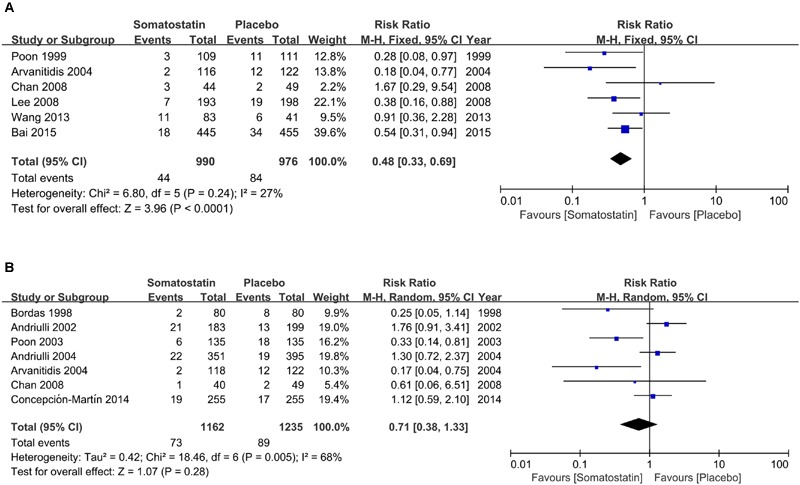
**Forest plot of RR of PEP when SOM was administrated at high (≥3000 μg) (A)** and low dose (<3000 μg) **(B)**. *I*^2^ and *P* is the criterion of heterogeneity test, ◆ pooled risk ratio, —■— risk ratio and 95% CI.

Subgroup analysis according to dose shows decreased incidence of PEP in patients treated with SOM at ≥3000 μg (4.44% vs. 8.61%) (RR = 0.48, 95% CI: 0.33, 0.69; *P* < 0.0001) (**Figure 4A**). However, when SOM dose decreased to less than 3000 μg, there is no significant difference in PEP risk (RR = 0.71, 95% CI: 0.38, 1.33; *P* = 0.28) (**Figure 4B**).

### Hyperamylasemia

Hyperamylasemia was analyzed in 10 studies and in a total of 4,032 patients. Hyperamylasemia occurred in 346 (16.7%) out of 2,072 patients treated with SOM, and 388 (19.80%) out of 1,960 patients treated with placebo (Supplementary Figure S2). The fixed-effect model demonstrated significantly decreased risk of hyperamylasemia (RR = 0.75, 95% CI: 0.66, 0.84; *P* < 0.001).

**FIGURE 5:**
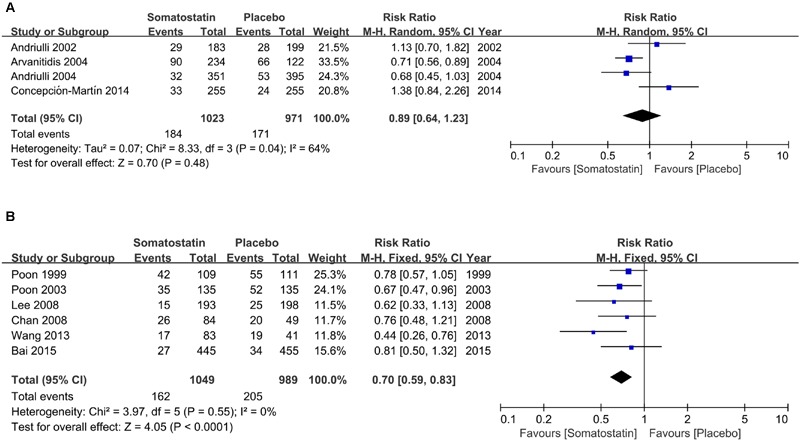
**Forest plot of RR of ERCP-induced hyperamylasemia in Europe (A)** and Asia **(B)**. *I*^2^ and *P* is the criterion of heterogeneity test, ◆ pooled risk ratio, —■— risk ratio and 95% CI.

Subgroup analysis according to ethnicity shows no difference in hyperamylasemia incidence in SOM and placebo-treated patients (17.99% vs. 17.61%) (RR = 0.89, 95% CI: 0.64, 1.23; *P* = 0.48) in Europe (**Figure 5A**). The incidence of hyperamylasemia was decreased in SOM patients (15.44% vs. 20.73%) (RR = 0.48, 95% CI: 0.34, 0.69; *P* < 0.001) in Asia (**Figure 5B**).

**FIGURE 6:**
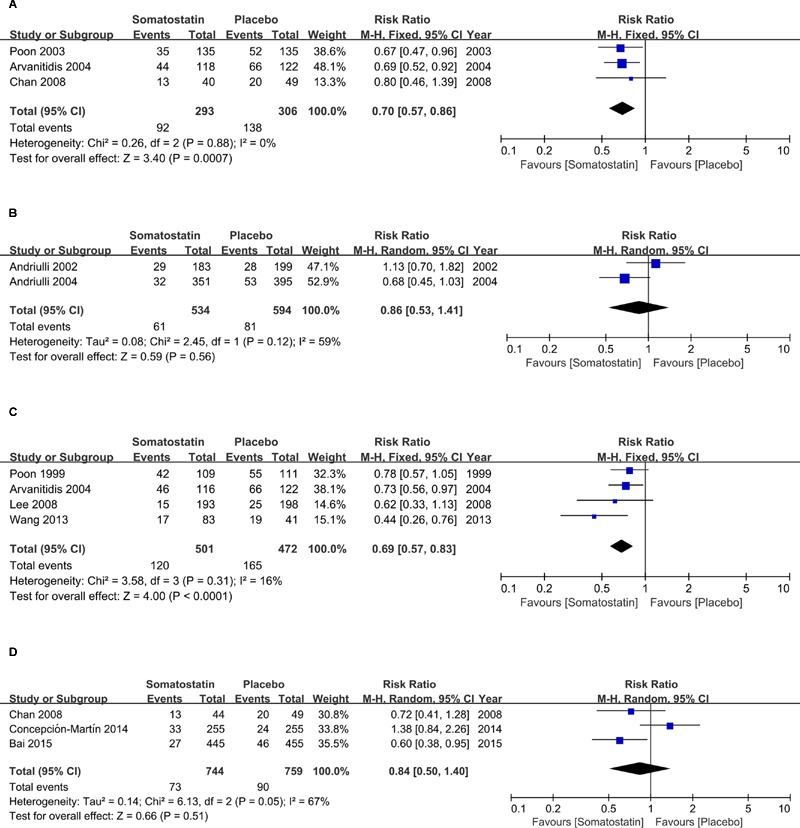
**Forest plot of RR of ERCP-induced hyperamylasemia in patients treated with SOM as single bolus (**A**)**, short-term infusion **(B)**, long-term infusion **(C)**, and bolus plus continuous infusion **(D)**. *I*^2^ and *P* is the criterion of heterogeneity test, ◆ pooled risk ratio, —■— risk ratio and 95% CI.

Subgroup analysis according to schedules showed decreased incidence of hyperamylasemia with a single bolus treatment of SOM compared with placebo (RR = 0.70, 95% CI: 0.57, 0.86; *P* < 0.001) (**Figure 6A**). When SOM was administrated as short-term infusion, the risk of hyperamylasemia incidence was not decreased compared with placebo (RR = 0.86, 95% CI: 0.53, 1.41; *P* = 0.56) (**Figure 6B**). We observed significantly decreased hyperamylasemia risk when SOM was administrated as long-term infusion (RR = 0.69, 95% CI: 0.57, 0.83; *P* < 0.0001) (**Figure 6C**), but no difference when SOM was treated as bolus plus continuous infusion (RR = 0.84, 95% CI: 0.50, 1.40; *P* = 0.51) (**Figure 6D**).

**FIGURE 7:**
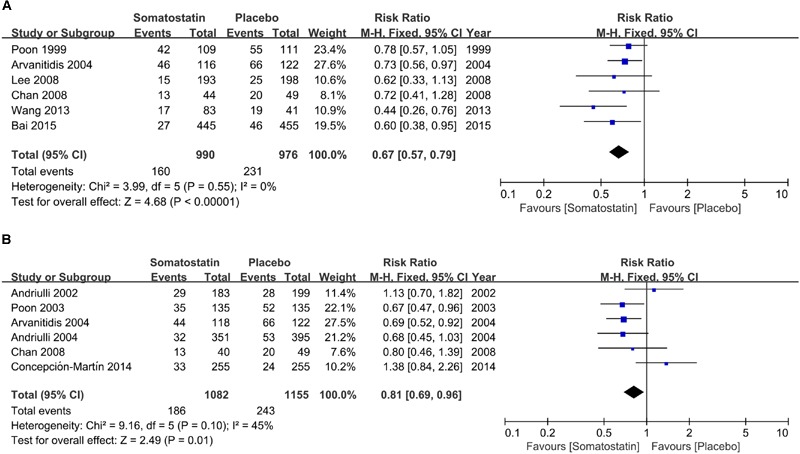
**Forest plot of RR of ERCP-induced hyperamylasemia SOM was administrated at high (≥3000 μg) (A)** and low dose (<3000 μg) **(B)**. *I*^2^ and *P* is the criterion of heterogeneity test, ◆ pooled risk ratio, —■— risk ratio and 95% CI.

Subgroup analysis according to dose shows decreased incidence of hyperamylasemia in patients treated with SOM at ≥3000 μg (16.16% vs. 23.67%) (RR = 0.67, 95% CI: 0.57, 0.79; *P* < 0.001) (**Figure 7A**). When SOM dose decreased to less than 3000 μg, we found significantly decreased risk of hyperamylasemia (RR = 0.81, 95% CI: 0.69, 0.96; *P* = 0.01) (**Figure 7B**).

### Abdominal Pain

Post-ERCP abdominal pain was reported in 163 (15.78%) out of 1,033 patients treated with SOM, and 168 (15.34%) out of 1,095 patients treated with placebo (Supplementary Figure S3). There is no significant difference in the risk of abdominal pain (RR = 0.89, 95% CI: 0.62, 1.27; *P* = 0.52).

### Publication Bias

**FIGURE 8:**
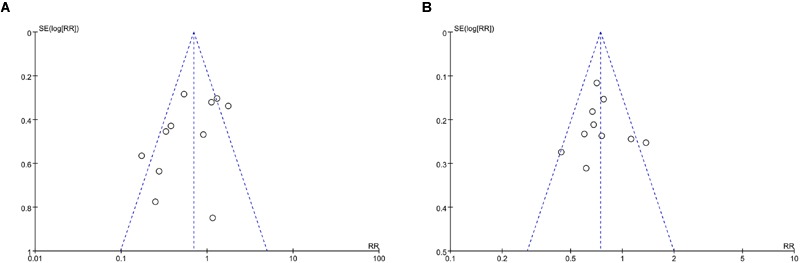
**Funnel plot for the publication bias. (A)** PEP. **(B)** hyperamylasemia.

A funnel plot was used to express the publication bias. There were 11 trials included in the funnel plot of incidence of PEP. A little asymmetry was observed in this funnel plot (**Figure 8A**). The funnel plot of hyperamylasemia was also applied. Publication bias was found in the outcome of hyperamylasemia (**Figure 8B**).

## Discussion

Despite medical condition enormously improved over the several decades, little progress has been made toward the goal of founding appropriate agents for preventing PEP (Kubiliun et al., 2015). SOM was firstly found potential for PEP in 1980s. However, the efficacy of SOM on PEP seems to be along with contradictory results based on several properly designed, well-executed, prospective randomized trials. Therefore, the opinion on its clinical benefit remains far from consistent. In recent years, there were mainly six meta-analyses focusing on the efficacy of SOM. Andriulli reported a meta-analysis of the preventive efficacy of somatostatin and its analog on PEP in Andriulli et al. (2000). The analysis concluded that pancreatic injury after ERCP could be prevented with the administration of SOM with an OR of 0.38 (95% CI: 0.22, 0.65; *P* < 0.001). Moreover, SOM was also able to reduce hyperamylasemia and abdominal pain (OR = 0.65, 95% CI: 0.48, 0.90; *P* = 0.008 and OR = 0.24, 95% CI: 0.14, 0.42; *P* < 0.001). After 7 years, Andriulli and Rudin respectively updated the meta-analyses by including several high-quality trials on SOM. The result from Andriulli’ research reported that SOM was ineffective in reducing PEP and pain. Meanwhile, there was limited efficacy on hyperamylasemia. The significant efficacy of SOM on PEP was obtained only in the subgroup of patients receiving with bolus injection (Andriulli et al., 2007). The research from Rudin also confirmed that SOM can significantly decrease the incidence of PEP with only long-term infusion or bolus. However, there was no difference between control and SOM arms with short-term infusion (Rudin et al., 2007). Further research performed by Omata summarized that the preventive efficacy of SOM was more prominent in cases high-dose administration over 12 h, or bolus injection (Omata et al., 2010). Kubiliun et al. (2015), Qin et al. (2015), two meta-analyses further confirmed that the benefit of SOM has been demonstrated more consistently with bolus administration than with infusion. Apart from bolus administration seemed to be the prominent method, the benefits from other administration methods, duration and dosage of SOM were inconsistent. In addition, two new clinical trials were further published from 2014 to 2015. We therefore conducted this meta-analysis to find out more accurate result of SOM on PEP.

Eleven high-quality RCTs involving a total of 4192 participants receiving SOM or placebo during ERCP were included in our study. The result showed a significant decline in incidence of PEP and hyperamylasemia based on the pooled data of SOM. It indicated that SOM might be effective on PEP. Further outcomes were analyzed to evaluate the preventive efficacy of subgroups according to area, treatment schedule and dosage. The pooled data indicated a remarkable decrease of PEP treated with SOM in Asia, whereas, there appeared no change between SOM and placebo treatment in Europe. The hyperamylasemia was in accordance with the change of PEP. Therefore, SOM might be more efficient for Asian than European. This is the first time to demonstrate the relationship between the efficacy of SOM and area. Moreover, we also explored the four subgroups according to schedule of treatment such as bolus, short-term infusion, long-term infusion and bolus plus continuous infusion to assess its preventive efficacy. SOM demonstrated significant decline of PEP and hyperamylasemia with both bolus and long-term infusion. The result is in according with previous study. Surprisingly, SOM was not effective applied with neither short-term infusion nor bolus plus continuous infusion. Moreover, SOM even presented the opposite trend to increase the incidence of PEP compared with placebo with short-term infusion. Why could single bolus be effective than short-term infusion? As a possible explanation, we supposed that single bolus of SOM was able to achieve the peak at a critical point which enabled SOM exert its protective efficacy of PEP. This critical point is possibly the introduction of the catheter to the papilla. On the contrary, the short-term infusion of SOM failed to yield the peak and resulted in ineffectiveness. The previous used to pointed out that there was a significant efficacy of SOM on PEP with high-dose over 12 h. We further investigated the relationship between dosage and efficacy. It was confirmed that SOM could decrease the incidence of PEP at no less than 3000 μg. In addition, SOM was also proved to be able to reduce hyperamylasemia in our study.

There are several limitations in our study. Firstly, we disregarded few non-English-language literatures. This might be one of the reasons for publication bias in our meta-analyses. Secondly, due to limited or missing data about subsets in current trials, there are still several details such as sex, age, reasons for ERCP and so on were unable to be analyzed in subgroups. Finally, the promising result of this study still need further confirmation by the most practical and likely cost-effective approach.

## Conclusion

The current meta-analytic data on efficacy of SOM on patients undergoing ERCP varied from area to dosage. It is clear from our study that the beneficial efficacy of SOM used in Asia was more likely to reduce the incidence of PEP and hyperamylasemia. Moreover, when given as a single bolus or long-term injection, SOM still maintains its role in this field. High dosage of SOM demonstrated the obvious efficacy than low dose. However, high-quality clinical trials are still needed to improve the residual doubts.

## Author Contributions

JH, P-LL, and TZ performed the search and contributed to manuscript writing. J-PC, Y-JH, and ZY contributed to data interpretation. P-LL and TZ performed the data extraction. J-PW, DZ, and X-FT amended the paper. JH designed the study and supervised the study operations. All authors read and approved the final manuscript.

## Conflict of Interest Statement

The authors declare that the research was conducted in the absence of any commercial or financial relationships that could be construed as a potential conflict of interest.

The reviewer FB and handling Editor declared their shared affiliation, and the handling Editor states that the process nevertheless met the standards of a fair and objective review.
