# Supplementary material for: Role of Somatostatin in Preventing Post-endoscopic Retrograde Cholangiopancreatography (ERCP) Pancreatitis: An Update Meta-analysis
Source: Front Pharmacol. 2016 Dec 15;7:489. doi: 10.3389/fphar.2016.00489 (PMC5156829; doi:10.3389/fphar.2016.00489)
Supplement: Supplementary file 1 [file Data_Sheet_1.DOC]

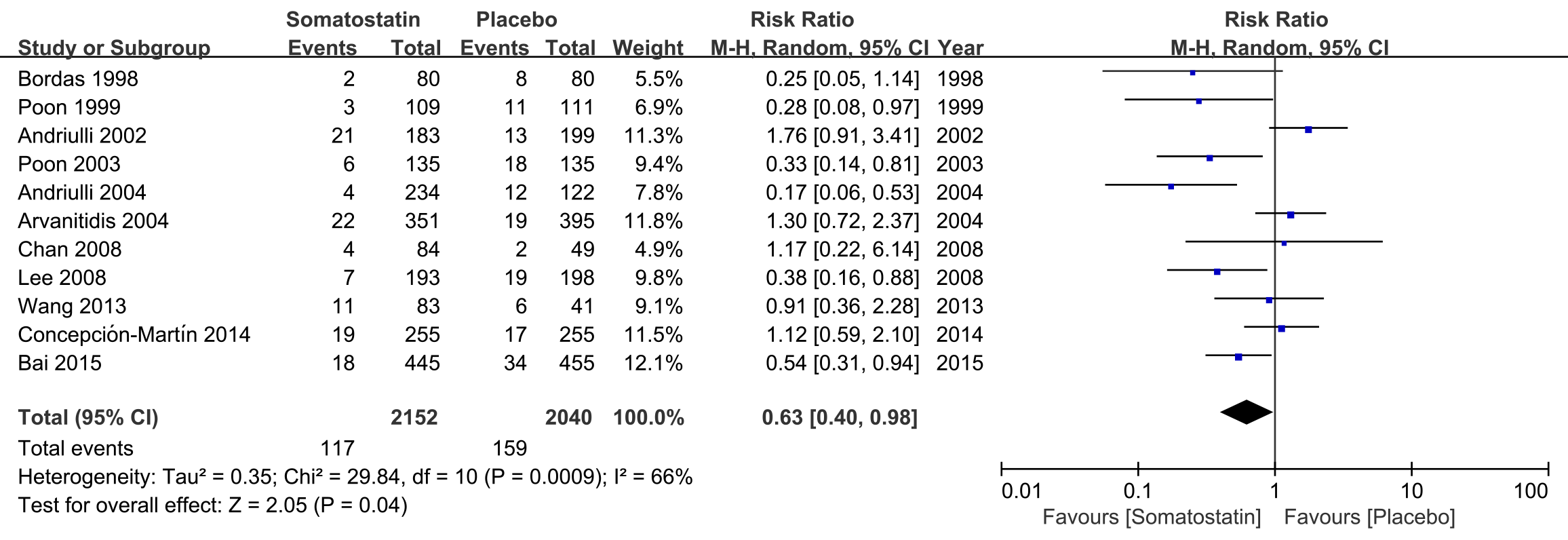


**Figure S1 The efficacy of SOM treatment on PEP compared with placebo.** *I*^2^ and *P* is the criterion of heterogeneity test, ◆ pooled risk ratio, —■— risk ratio and 95% CI.


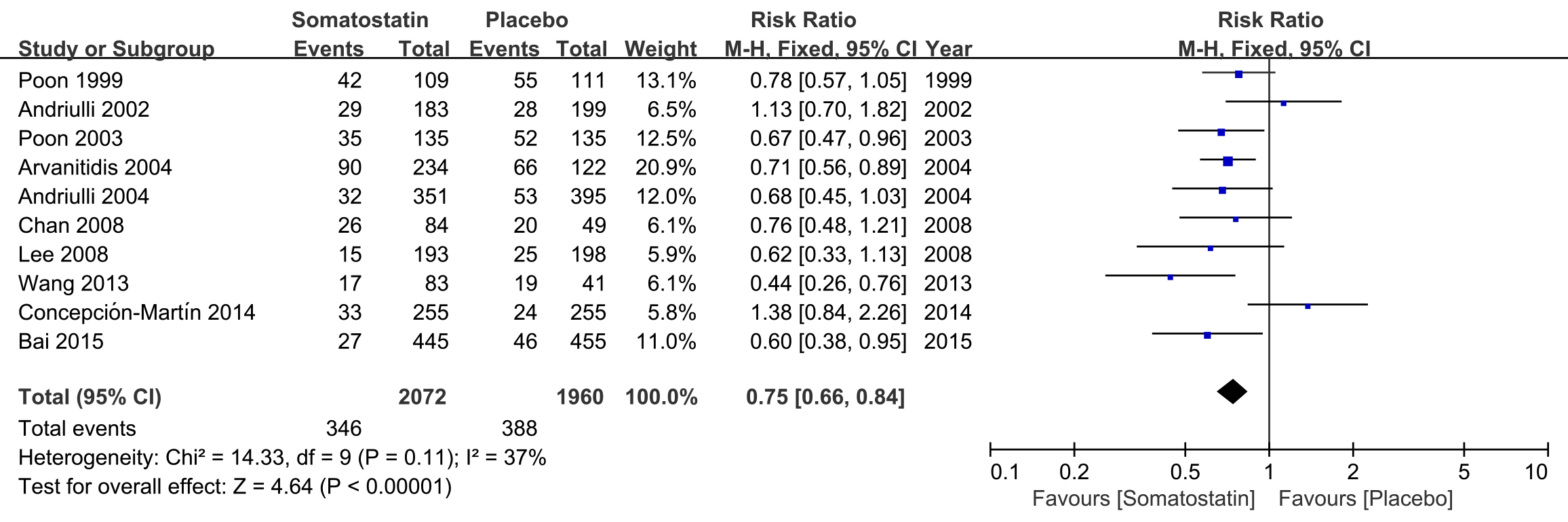


**Figure S2 The incidence of** **ERCP induced hyperamylasemia on SOM treatment compared with placebo.** *I*^2^ and *P* is the criterion of heterogeneity test, ◆ pooled risk ratio, —■— risk ratio and 95% CI.


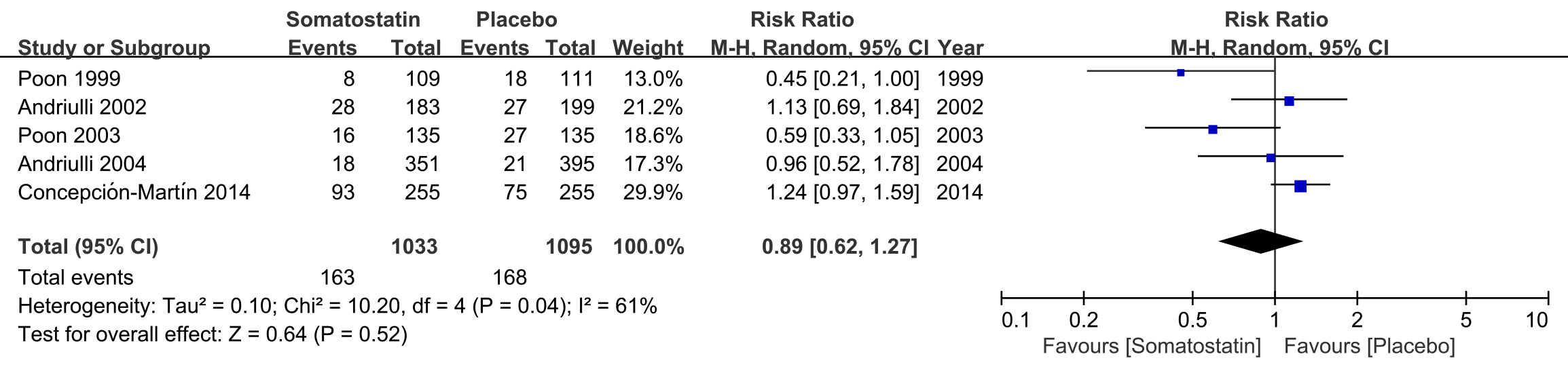


**Figure S3 The incidence of ERCP induced abdominal pain on SOM treatment compared with placebo.** *I*^2^ and *P* is the criterion of heterogeneity test, ◆ pooled risk ratio, —■— risk ratio and 95% CI.

**Table S1 Baseline characteristics of the included trials.**

| Study  (years) | Country | Study design | Treatment | Number of Patients | Age (years)  Range,mean | Male/Female | Jadad quality score |
| --- | --- | --- | --- | --- | --- | --- | --- |
| Bordas 1998 | Spain | RCT | SOM | 80 | 61.0±15.1  58.0±17.5 | 45/35  51/29 | 4 |
|  |  |  | Placebo | 80 |  |  |  |
| Poon 1999 | Hong Kong | RCT | SOM | 109 | 62.8±15.1 | 52/57 | 5 |
|  |  |  | Placebo | 111 | 62.7±16.1 | 50/61 |  |
| Andriulli 2002 | Italy | RCT | SOM | 183 | 59.0±18.0 | 91/92 | 5 |
|  |  |  | Placebo | 199 | 58.0±17.0 | 91/108 |  |
| Poon 2003 | Hong Kong | RCT | SOM | 135 | 69 (56-76)  67 (52-75) | 65/70  72/63 | 5 |
|  |  |  | Placebo | 135 |  |  |  |
| Andriulli 2004 | Italy | RCT | SOM | 351 | 66±15 | 177/174 | 5 |
|  |  |  | Placebo | 395 | 66±16 | 220/175 |  |
| Arvanitidis 2004 | Greece | RCT | SOM | 118 | 65±13 | 72/46 | 4 |
|  |  |  | SOM | 116 | 63±13 | 74/42 |  |
|  |  |  | Placebo | 122 | 61±12 | 66/56 |  |
| Chan 2008 | Taiwan | RCT | SOM | 44 | 59.7±15.5  62.9±14.5 | 26/18  20/20 | 5 |
|  |  |  | SOM | 40 |  |  |  |
|  |  |  | Placebo | 49 | 65.4±13.7 | 26/23 |  |
| Lee 2008 | Korea | RCT | SOM | 193 | 63±14 | 100/93 | 4 |
|  |  |  | Placebo | 198 | 62±14 | 100/98 |  |
| Wang 2013 | China | RCT | SOM | 36 | 58.7±16.0  64.6±16.8 | 20/16  21/16 | 4 |
|  |  |  | SOM | 47 |  |  |  |
|  |  |  | Placebo | 41 | 61.2±13.6 | 20/21 |  |
| Concepción-Martín 2014 | Spain | RCT | SOM | 255 | 73±14 | 116/139 | 5 |
|  |  |  | Placebo | 255 | 73±13 | 125/130 |  |
| Bai 2015 | China | RCT | SOM | 445 | 60 (19-96) | 197/248 | 5 |
|  |  |  | Placebo | 455 | 63 (22-93) | 237/218 |  |

RCT, Randomized controlled trial; SOM, Somatostatin.
